# Dermatoscopic Patterns in Mycosis Fungoides: Observations from a Case-Series Retrospective Analysis and a Review of the Literature

**DOI:** 10.3390/diagnostics15091136

**Published:** 2025-04-29

**Authors:** Corrado Zengarini, Federica Tugnoli, Alessio Natale, Martina Mussi, Giacomo Clarizio, Claudio Agostinelli, Elena Sabattini, Alberto Corrà, Bianca Maria Piraccini, Alessandro Pileri

**Affiliations:** 1 Department of Medical and Surgical Sciences, University of Bologna, 40138 Bologna, Italyalessandro.pileri2@unibo.it (A.P.); 2 Dermatology Unit, IRCCS Azienda Ospedaliero-Universitaria di Bologna, 40138 Bologna, Italy; 3 Haematopathology Unit, IRCCS Azienda Ospedaliero-Universitaria di Bologna, 40138 Bologna, Italy; 4 Dermatology Unit, Ospedale San Bartolo, 36100 Vicenza, Italy

**Keywords:** MF, dermoscopy, microscopy, non-invasive, diagnosis, patterns, CTCL

## Abstract

**Background:** Dermoscopy, a non-invasive diagnostic technique, is being increasingly used to evaluate cutaneous T-cell lymphomas such as mycosis fungoides (MF) and Sézary syndrome (SS). However, its diagnostic accuracy and role in staging remain underexplored. **Objective:** This study aimed to assess the dermoscopic patterns in MF and SS, correlating the findings with the disease stage and lesion type to evaluate dermoscopy’s diagnostic utility. **Methods:** A retrospective, monocentric analysis was conducted on patients with histologically confirmed MF or SS. Dermoscopic images were evaluated for vascular patterns, pigmentation, scaling, and keratin plugs. The statistical analysis assessed the correlations between these dermoscopic features and the TNMB staging and lesion type. A literature review was also performed to contextualize the findings, focusing on studies describing dermoscopic features in MF based on retrospective, prospective, and cross-sectional data. **Results:** The study included 30 patients with histologically confirmed MF or SS (19 males and 11 females; mean age: 64.5 years). The dermoscopic evaluation revealed that all the lesions were pigment-free, with vascular structures as the predominant feature. Linear vessels (40%) and serpentine vessels (13.3%) were the most frequently observed, along with dotted vessels (36.7%) and clods (10%). The vessel distribution was diffuse (40%) or perifollicular (36.7%), with a predominant red (56.7%) or orange (40%) background. Scaling was present in 76.7% of cases, either diffuse (40%) or perifollicular (36.7%), and keratin plugs were detected in 40% of the lesions. No statistically significant correlations were found between dermoscopic features and the TNMB stage or lesion type (*p* > 0.05). A cluster analysis identified two patient groups with differing vascular and scaling features but no clear association with disease stage. The literature review identified studies that commonly reported features in MF dermoscopy, including fine, short linear vessels and an orange-yellow background, particularly in early-stage MF. Spermatozoa-like structures have been marked as highly specific for diagnosing MF. Some studies also suggested a transition in vascular morphology from linear vessels in early disease to branched vessels and ulceration in advanced stages. **Conclusions:** Our results showed some vascular patterns have some potential but lack sensitivity for staging MF and SS. The terminology used and the reproducibility of our results compared to those reported in the literature showed little consistency, with none of our cases showing spermatozoa-like structures. Moreover, the same issues with the use of non-reproducible terminology were noted across the studies because it is not standardized and due to different incongruent dermoscopic patterns. More significant prospective studies with standardized descriptors and larger groups are needed to refine its diagnostic and staging utility.

## 1. Introduction

Primary cutaneous lymphomas (PCLs) are a subset of non-Hodgkin lymphomas (NHLs) that are characterized by a monoclonal proliferation of malignant lymphocytes within the skin [1]. Around three-quarters of these cases fall under the category of cutaneous T-cell lymphomas (CTCLs), with mycosis fungoides (MF) and Sézary syndrome (SS) being the most common subtypes [2].

Classic MF, first detailed by Jean Alibert and Ernest Bazin, typically begins with an early phase marked by scaly, erythematous patches or thin plaques of varying shapes and sizes, often appearing in sun-protected regions. Over time, the disease may evolve into an advanced stage, frequently characterized by the development of tumors [3]. On the other hand SS is a rare and aggressive leukemic variant of CTCL, distinct from MF, and is characterized by erythroderma, lymphadenopathy, and leukemic involvement of the peripheral blood [4,5]. Although MF and SS are the most well-known forms of CTCL and were once considered part of the same disease spectrum, they exhibit distinct clinical, pathological, and pathogenetic features. For instance, MF typically begins with localized patches or plaques and follows an indolent course, while SS shows a more aggressive behavior [6]. From a pathogenetic perspective, emerging evidence suggests that MF and SS may represent biologically distinct entities rather than different stages of the same disease; recognizing these differences is essential for accurate diagnosis and management.

CTCLs are rare, with an estimated annual incidence of 0.5 to 1 case per 100,000 individuals [7,8,9,10] and a prevalence of over 6 cases per 100,000 in Europe [11]. It primarily affects adults, with a median age of onset between 55 and 60 years, and a slight male predominance. The incidence rates vary geographically, being higher in Western countries than in Asian populations, likely due to genetic and environmental factors [12]. While the exact etiology remains unclear, potential risk factors include chronic antigenic stimulation, immune dysregulation, infections with Human T-Lymphotropic Virus type 1 (HTLV-1) or Epstein–Barr Virus (EBV), and genetic predispositions. Exposure to pesticides, industrial solvents, and environmental toxins may increase the risk, although a definitive causal relationship has not been established.

The biochemical developments of CTCL are complex, involving specific cytokines that regulate the inflammatory response and that vary across different disease stages, influencing its dynamics. Specifically, early-stage MF is characterized by a Th1-dominant cytokine profile, involving pro-inflammatory mediators such as IFN-γ, TNF-α, and IL-12 which play key roles. These cytokines drive the recruitment of T-cells and macrophages, leading to chronic inflammation and tissue damage and as the disease progresses, there is a shift toward a Th2 cytokine profile, marked by increased production of IL-4, IL-5, and IL-13 [13,14]. This change promotes tumor cell survival and suppresses effective immune responses, contributing to the persistence and spread of malignant cells. The interaction between these cytokine profiles drives the inflammatory microenvironment in MF, resulting in cutaneous histopathological alterations that often resemble other dermatoses. Consequently, clinical manifestations such as erythematous patches, plaques, and scaling can be misleading, making differential diagnosis challenging without specific immunohistochemical and molecular studies.

The diagnosis of MF relies on a combination of clinical, histological, and immunophenotypic criteria given its diverse presentations and overlap with inflammatory and neoplastic conditions [7]. Histological evaluation remains essential for confirming the diagnosis, assessing the disease phase, and classifying the MF subtypes, but early-stage MF often mimics a broad spectrum of reactive dermatoses and cutaneous lymphoproliferative disorders, making a definitive diagnosis and also therapy challenging [8,15]. In the earliest phases, histopathological findings may be subtle and non-specific, leading to delays in diagnosis or misclassification. This is especially true in patch-stage MF, where distinguishing it from chronic dermatitis, psoriasis, or parapsoriasis can be challenging.

To improve the diagnostic accuracy for early MF, the International Society for Cutaneous Lymphomas (ISCL) has developed a diagnostic algorithm that integrates clinical, histopathological [16], and immunohistochemical findings, along with T-cell receptor (TCR) gene rearrangement studies in cases with equivocal features [17]. This structured approach aims to enhance diagnostic specificity by incorporating molecular and immunophenotypic parameters, particularly in borderline cases where histological findings alone may be insufficient.

However, while the ISCL algorithm has demonstrated diagnostic utility, several studies have reported its specificity and accuracy limitations, especially in distinguishing early MF from inflammatory mimickers. Overlap in histological patterns, variability in TCR gene rearrangement analyses, and differences in interpretation across pathologists contribute to diagnostic uncertainty. Additionally, the algorithm does not fully account for the dynamic progression of MF, meaning that longitudinal clinical and histological follow-up remains crucial in ambiguous cases.

Given these challenges, non-invasive diagnostic tools, such as dermoscopy, have gained attention as potential adjunctive methods to support early detection and differentiation of MF. By providing real-time visualization of vascular, pigmentary, and scaling patterns, dermoscopy may offer additional insights into the morphological characteristics of MF lesions, helping to bridge the gap between clinical and histopathological evaluation [18].

Dermoscopy, a non-invasive diagnostic technique initially developed for assessing pigmented lesions, has expanded its use to various dermatological conditions, including cutaneous lymphoproliferative disorders like MF [19]. By visualizing subsurface skin structures, dermoscopy bridges clinical and histopathological evaluations, revealing features like vascular changes and scaling that may help distinguish MF from benign dermatoses. Dermoscopic evaluation is integrated into the standard diagnostic workflow, combining patient history and clinical examination to assess lesion number, distribution, and morphology. As its application broadens, dermoscopy continues to evolve, contributing to the diagnostic framework for various skin disorders, including those previously not considered within its scope [20].

Despite its potential, the diagnostic accuracy of dermoscopy in MF remains debated, with studies reporting inconsistent findings.

This study evaluated dermatoscopic patterns observed in patients with histologically confirmed MF and SS. Specifically, this research sought to determine whether certain dermatoscopic features correlate with disease stage or clinical presentation, potentially enhancing the utility of dermoscopy in the diagnostic workup of CTCLs. Therefore, this study will test the above criteria regarding standard diagnostic accuracy measures (DAMs) and inter-observer reproducibility.

## 2. Material and Methods

This study was conducted as an observational, monocentric, retrospective analysis at the specialized cutaneous lymphoma clinic of the Dermatology Department of Sant’Orsola-Malpighi Polyclinic in Bologna. The patients included in the study had histologically confirmed diagnoses of MF or SS and were treated between January and December 2019. All patients provided written consent, and the study was approved by the local Institutional Review Board (protocol code: CL.ClinIstoTp.19).

### 2.1. Patient Selection

The inclusion criteria were as follows: (1) histologically confirmed diagnosis of MF or SS, (2) available high-quality dermatoscopic images across multiple disease stages, and (3) follow-up data are sufficient to assess disease progression. Patients were excluded if they had incomplete clinical records or dermatoscopic images of insufficient quality. High-quality dermatoscopic images were defined as in-focus images with a minimum resolution of 96 dpi, without visible distortions or artefacts. Only images meeting these criteria were included in the analysis.

### 2.2. Dermatoscopic Evaluation

Dermatoscopic images were obtained using digital dermatoscopy with magnifications of up to 40×. All the photos were standardized with consistent lighting conditions and uniform acquisition protocols. The analysis focused on specific dermatoscopic features, including the presence or absence of pigmentation, vessel morphology (e.g., linear, serpentine, clod, or dotted vessels), vessel distribution patterns (e.g., clustered or serpiginous), background coloration, scaling characteristics, and the type and presence of keratin plugs.

### 2.3. Images Evaluations

Two experienced dermatologists independently evaluated all the dermatoscopic images using standardized dermatoscopic terminology. The features assessed included pigmentation, vessel morphology, vessel distribution, presence and type of keratin plugs, desquamation characteristics, distribution, and background coloration. The variables analyzed were based on those proposed by the International Dermatology Society consensus for oncological manifestations [21,22].

### 2.4. Statistical Analysis

The dermatoscopic features were correlated with clinical stages of MF/SS using the EORTC staging [17]. Statistical analysis was conducted using the Mann–Whitney U test to evaluate the associations between dermatoscopic features and the TNMB stage. The analyzed features included vessel morphology, distribution, background coloration, scaling, and keratin plugs. The analysis aimed to identify potential correlations between these dermatoscopic characteristics, TNMB stages, lesion types (patches, plaques, and nodules), and disease progression.

An automatic two-step cluster analysis was also performed to identify patterns within the dermatoscopic features that are potentially associated with specific lesion types or disease stages. The data analysis was carried out using SPSS software (version 26.0), with descriptive statistics, including means, standard deviations, and frequencies, used to summarize the dermatoscopic features across the clinical stages. Graphical data representation was generated using Jupyter Notebook (Python, ver. 7, available on https://jupyter.org/), with custom scripts developed for data visualization.

### 2.5. Literature Review and Article Selection

For a synthetic narrative review of the literature, manuscript searches were conducted on PubMed, Scopus, and Web of Science to identify studies on the dermoscopic features of MF and SS published between 2013 and 2025. Articles were included if they analyzed histologically confirmed cases, correlated dermoscopy with disease stage, and included at least 10 patients.

The key dermoscopic features were assessed, described, and discussed in the context of our results.

## 3. Results

**Figure 1:**
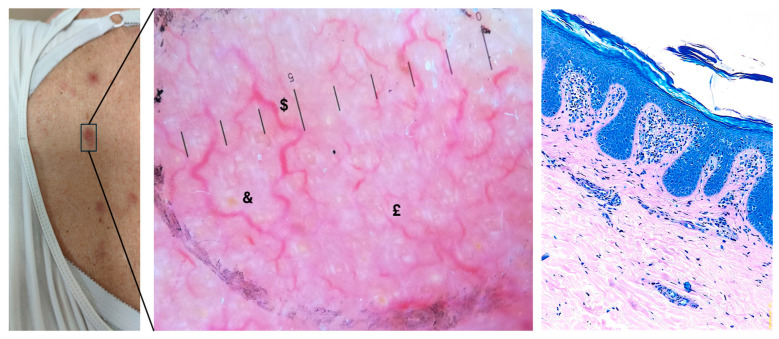
Macroscopic, dermoscopic, and histological examination of a suspicious cutaneous lesion (10× magnification). On the left, a clinical image shows an erythematous lesion on the patient’s abdomen, with poorly defined margins and mild surface scaling. In the central figure, histological analysis of the lesion stained with Giemsa reveals epidermotropic, hyperchromatic lymphocytes alongside a band-like lymphoid infiltrate in the dermis, set against a background of psoriasiform acanthosis and dermal fibrosis. On the right, a magnified dermoscopic image reveals a predominant vascular pattern, featuring branched vessels ($) surrounding white and yellow keratin plugs, along with serpiginous vessels (£) on a heterogeneous erythematous background (&). The presence of subtle scaling may suggest concurrent inflammation (10× magnification).

The study included 30 patients with histologically confirmed mycosis fungoides (MF) or Sézary syndrome (SS), comprising 19 males and 11 females, with a mean age of 64.5 years (range: 32–93). The patients presented with clinical lesions, including patches, plaques, and nodules, spanning various TNMB stages, from early-stage I A to advanced-stage IV A 1 (Table 1). Dermoscopic examination commonly revealed non-pigmented MF lesions, predominantly exhibiting linear and serpiginous vascular patterns, occasionally with perifollicular distribution (Figure 1). While these dermoscopic features were recurrent, no consistent correlation was observed between vascular patterns (except for serpentine vessels), the TNMB stage, or the specific lesion type (Table 2).

### 3.1. Dermatoscopic Findings

**Figure 2:**
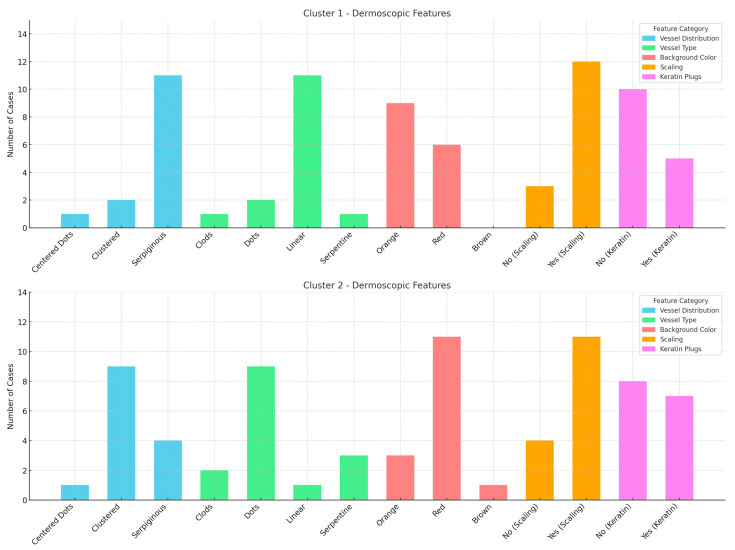
Graphs of the dermoscopic features for two distinct clusters, showing the distribution of vessel type, vessel distribution, background color, scaling, and keratin plugs across the two groups. Cluster 1 and 2 are differentiated by their dermoscopic patterns, with noticeable variations in vessel types and background colors.

Across all patients, 100% of the lesions were pigment-free, and blood vessels were visible in every lesion. Vascular structures were the most prominent dermatoscopic feature, with linear vessels observed in 12 cases (40%) and serpentine vessels in 4 cases (13.3%). Other vessel morphologies included dotted vessels (11 cases, 36.7%) and clods (3 cases, 10%). The vessel distribution patterns varied, with 12 lesions (40%) showing a diffuse distribution and 11 (36.7%) showing perifollicular distribution. The predominant background color was red, observed in 17 cases (56.7%), followed by orange in 12 cases (40%) and, in a minority of cases, brown (1 case, 3.3%). Scaling was observed in 23 patients (76.7%), with equal distribution between diffuse scaling (12 cases, 40%) and perifollicular scaling (11 cases, 36.7%). Keratin plugs were present in 12 cases (40%); when observed, they were predominantly yellow (Table 2, Figure 2).

### 3.2. Correlation with TNMB Stages and Lesion Types

Fisher’s exact tests were performed to evaluate the associations between dermoscopic features and both lesion type and diagnosis. While most features, including vessel presence, scaling distribution, and background color, showed no significant associations, vessel morphology was significantly related to diagnosis (*p* = 0.002). Specifically, linear vessels were predominantly observed in classic mycosis fungoides, serpentine vessels in folliculotropic MF, and clods in both classic MF and Sézary syndrome (Table 3).

However, the Mann–Whitney U test failed to demonstrate any statistically significant correlation between individual dermatoscopic features and TNMB stage. All *p*-values for vessel type, vessel distribution, background color, scaling, and keratin plugs were higher than 0.05, suggesting no strong association between these dermatoscopic patterns and disease stage. Similarly, no significant relationship was found between specific dermatoscopic features and lesion types (patch, plaque, or nodule).

**Figure 3:**
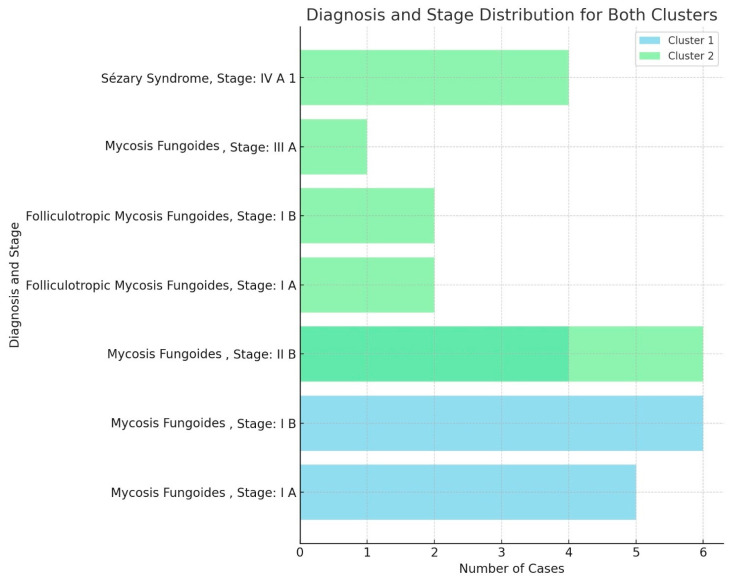
Panel providing diagnosis and TNMB stage distribution for both clusters, highlighting the representation of mycosis fungoides and Sézary syndrome cases across the various stages (I A, I B, II B, III A, and IV A 1). The cases for each diagnosis and stage were compared between the two clusters.

The cluster analysis identified two patient groups: Cluster 1, characterized by the presence of dots, clods, and less than 50% linear vessels, with no serpiginous distribution, predominantly associated with nodular lesions, and Cluster 2, characterized by serpentine and linear vessels with a serpiginous distribution, which were observed in lesions at various stages. However, these clusters did not show significant subgroups nor a possible correlation with TNMB staging (Figure 3).

### 3.3. Literature Review

Nine studies, including 411 patients, published between 2013 and 2025, examined the role of dermoscopy in diagnosing and staging mycosis fungoides (MF) [23,24,25,26,27,28,29,30,31]. These studies analyzed the dermoscopic features across different MF subtypes, variations in vascular and pigmentary structures based on skin tone, and correlations with histopathological findings.

#### 3.3.1. Dermoscopy in Early-Stage MF

Most studies reported that fine, short linear vessels, spermatozoa-like structures, and an orange-yellow background are the most frequently observed dermoscopic markers in early-stage MF, particularly in the patch stage [14]. These features were described as having high sensitivity and specificity in differentiating patch-stage MF from clinically similar dermatoses, including eczema, psoriasis, and subacute cutaneous lupus erythematosus (SCLE) [15].

Repetitive dermoscopic patterns were identified in patch-stage MF, consisting of a non-homogeneous pink to the erythematous background, patchy orange-red discoloration, white scaling, and a combination of dotted, short linear, and spermatozoa-like vessels [15]. The presence of spermatozoa-like vessels, defined as a mixture of linear and dotted vascular patterns, was consistently noted across studies [15]. The vascular distribution was irregular and patchy, in contrast to the more uniform dotted vessel pattern observed in psoriasis [16].

Histopathological analyses showed that linear vessels correlated with elongated and dilated capillaries in the papillary dermis, while the orange-yellow background was associated with epidermal atrophy and dermal fibrosis [16]. The white scaling observed in MF lesions corresponded to hyperkeratosis and focal parakeratosis [16].

#### 3.3.2. Vascular and Scaling Variability in Disease Progression

Several studies reported a correlation between vascular morphology and MF disease progression. Fine linear vessels were predominant in patch-stage MF, while in plaque and tumor-stage MF, branched linear vessels, ulceration, and bright white structureless areas were more frequently observed [17]. Bright white areas were mainly present in advanced lesions, corresponding histologically to dermal fibrosis and sclerotic changes [17].

The scaling patterns varied across the different MF stages. Patch-stage lesions were associated with fine, patchy white scales, whereas thicker, perifollicular, or geometric white scales were more commonly observed in plaque-stage MF [18]. Geometric white scales were histologically correlated with alternating parakeratosis and orthokeratosis in the stratum corneum [18].

Several studies analyzed vascular and scaling patterns in terms of TNMB staging, identifying a transition from linear vessels in early MF to branched vascular structures in more advanced disease stages [19]. Differences in background coloration, vascular morphology, and scaling type were also noted across MF stages [20]. 

#### 3.3.3. Dermoscopic Features in MF Subtypes and Variants

Other studies analyzed the dermoscopic differences across MF subtypes. In stage IIA MF, the most frequently observed features were orange-yellow patches (88.2%), short fine linear vessels (82.3%), geometric white scales (70.5%), perifollicular white scales (47%), and white patches (35.2%) [21]. In contrast, in parapsoriasis (PP), dotted vessels (94.1%), diffuse lamellar white scales (88.2%), and dotted and globular vessels (70.5%) were more frequently reported [21]. Additional findings such as spermatozoa-like structures, purpuric dots, collarette white scales, and Y-shaped arborizing vessels were identified in MF but were not statistically significant [21].

In folliculotropic MF, studies consistently reported follicular keratotic plugs and perifollicular white scaling as the most characteristic dermoscopic findings [22]. In addition to follicular changes, dilated follicles and lack of hair structures were also observed, corresponding histologically to follicular infiltration by neoplastic T-cells [22,23,32].

One study analyzed dermoscopic features in purpuric MF and found that fine, short linear vessels and spermatozoa-like structures were significantly more common in MF, whereas pigmented purpuric dermatoses (PPDs) showed erythematous globules, reticular pigmentation, and a dull red background [24].

A study on MF in skin of color identified white streaks and a pseudo network pattern as predominant dermoscopic features, with vascular morphology playing a less significant role compared to findings in lighter skin tones [25].

Hair shaft abnormalities, particularly pili torti and 8-shaped hairs, were described as a novel dermoscopic finding in patch/plaque-stage MF [26]. These features were suggested to correlate with follicular involvement in the disease [26].

#### 3.3.4. Artificial Intelligence in CTCL Diagnosis: Emerging Insights and Future Directions

In recent years, artificial intelligence (AI) has emerged as a transformative tool in dermatology, particularly in the diagnosis and management of skin malignancies [33]. Convolutional neural networks (CNNs) and other machine learning algorithms have demonstrated proficiency in classifying skin lesions, often achieving diagnostic accuracies comparable to, or exceeding, those of experienced dermatologists [34].

Beyond melanoma and non-melanoma skin cancers [35,36,37,38,39], AI applications have expanded to include adnexal tumors and inflammatory skin conditions, utilizing both clinical and dermoscopic imaging [40,41,42,43]. In wound care, AI-driven models have been employed to assess and monitor healing processes, leveraging various imaging techniques and mobile-based devices to provide timely and accurate evaluations [44,45,46,47]. These advancements have facilitated personalized treatment strategies and improved patient management across diverse dermatological conditions.

Despite these successes, the integration of AI into the evaluation of CTCLs remains limited since they present with heterogeneous clinical and histopathological features, posing challenges for standardized image analysis. Studies have emphasized the nascent stage of AI applications in this domain, suggesting that AI could assist in digital pathology for skin cancers and lymphomas by automating diagnostic processes, quantifying biomarkers, and predicting outcomes [48,49].

There have been studies assessing AI performance in detecting CTCLs from clinical and dermoscopical imaging which showed some results but underscored the need for comprehensive research to validate AI models in this context; some authors also advocated for systematic reviews and meta-analyses to evaluate the diagnostic accuracy and clinical utility of AI in lymphoma detection [48,50].

In the end, the limited application of AI in cutaneous lymphomas may be attributed to several factors, including the rarity of these conditions, the complexity of their presentation, and the scarcity of large, annotated datasets necessary for training robust AI models and, to date and above all, the lack of consensus on the terms predominantly used for dermoscopic annotation. Addressing these challenges requires collaborative efforts to curate extensive image repositories and develop algorithms capable of capturing the nuanced features of lymphoproliferative disorders.

AI has potential in various areas of dermatology, but its application in the diagnosis and management of cutaneous lymphomas remains mostly underexplored and hard to assess.

#### 3.3.5. Study Limitations and Summary

All the reviewed studies suffered from different degrees of selection bias due to study design [26]. Most studies had small sample sizes, and many were single-center retrospective analyses [26]. Additionally, standardized criteria for dermoscopic evaluation were sometimes lacking, making a direct comparison across studies difficult [26]. Variability in image acquisition techniques, magnifications, and dermoscopic terminology further contributed to inconsistencies in the reported findings [27].

Despite these limitations, the studies provide insights into recurrent dermoscopic features across MF subtypes, including variations based on the disease stage, skin tone, and lesion type [28]. The findings from the reviewed studies are summarized in Table 4.

## 4. Discussion

MF and SS are among the primary lymphoid-derived cancers of the skin [51], representing the most common subtypes of CTCLs.

A broad spectrum of clinical appearances and significant variability among different patients characterizes MF. Apart from the most frequent clinical pictures, it may also encompass bullous, hypopigmented, ichthyosiform, keratoderma-like, pigmented purpuric dermatosis-like, papular, poikilodermatous, psoriasiform, pustular, and verrucous morphologies [3,29]. The wide array of presentations can make the diagnosis of MF particularly challenging since it often mimics other dermatological conditions, thus expanding the extensive differential diagnoses, which include both benign inflammatory dermatoses and malignant diseases. Moreover, it is of note that, in early stages, MF often shows subtle, non-specific histopathological findings, requiring a careful process of correlation with immunohistochemical features to reach a definitive diagnosis [16].

Alongside the challenges in achieving accurate MF identification, the frequent diagnostic delay in the early phase is another significant issue. Initial lesions are often misclassified as benign inflammatory conditions like eczema or psoriasis, resulting in extended treatments without a previous histological evaluation, possibly hesitating in the rapid progression of the cutaneous lymphoma [52,53]. The early diagnosis of MF can significantly impact the final treatment outcomes by enabling the use of mild, non-harmful, skin-directed therapies, leading to better clinical results and quality of life [52]. Therefore, any method, tool, or approach showing a potential contribution to improving the accuracy and reliability of the diagnostic process should be embraced and thoroughly studied to ensure its efficacy and applicability.

Similar to other dermatological conditions, significant efforts are being made to integrate dermoscopy into the diagnostic workflow for MF and SS to improve the accuracy of the clinical evaluation. As highlighted by various authors, dermatoscopy has become an indispensable tool in modern practice, often referred to as the dermatologist’s stethoscope. Dermoscopy may provide valuable diagnostic support by identifying specific patterns that correspond to histopathological alterations in the examined tissue, allowing for a noninvasive and highly informative assessment.

Our results, however, showed no significant correlations found between dermoscopic patterns and the lesion type or TNMB staging except for serpentine vessels being more predominant in the FMF subtype. However, this needs to be cautiously considered due to low reliability of the test. Considering the inherent complexity of MF diagnosis due to the clinical and histological features closely resembling those of other skin conditions, it is unlikely that dermoscopic characteristics alone would be highly specific enough to significantly narrow the differential diagnosis.

An orange-erythematous background is a well-documented dermoscopic feature of the patch stage in MF, as supported by previous studies [23,24]. Nevertheless, this characteristic is not exclusive to MF, as it is also commonly seen in inflammatory dermatoses such as granulomatous inflammatory or infectious diseases [54,55], cutaneous xanthomas, and pityriasis rubra pilaris [56].

Similarly, the type and distribution of scales observed in our MF cases align with findings from earlier research [24,27]. However, these features are also detectable in a broad spectrum of inflammatory skin disorders, limiting their specificity for MF diagnosis.

In contrast, the literature highlights specific dermoscopic features in early-stage MF, particularly regarding vascular morphology. Previous studies have identified fine short linear vessels as a highly sensitive (93.7%) and specific (97.1%) dermoscopic feature of early-stage MF [19,21,24,26] compared to chronic dermatitis, which represents one of the most common differential diagnoses. Moreover, detecting a linear disposition of blood vessels was advocated to be more associated with the early stage of the disease. In contrast, dotted vessels and red clods seemed to correlate with later phases of the disease, possibly due to the vertical growth pattern of skin lesions [29]. Polymorphous vascular structures, on the other hand, are more commonly reported in CD30+ anaplastic large-cell lymphoma [25,26,57].

However, our findings, as well as those of other studies in the literature, did not demonstrate a clear and robust correlation between vessel type and disease stage, suggesting that dermoscopy alone is insufficient for staging MF or SS. Our study’s absence of consistent patterns emphasizes the necessity of histopathological confirmation in diagnosing cutaneous lymphoproliferative disorders (CLDs) and CTCLs.

These results support the need for additional research to better define the potential diagnostic role of dermoscopy in early MF detection [58]. Even in the absence of high diagnostic specificity, the description of dermoscopic patterns of MF may increase clinician awareness and potentially lead to a reduced diagnostic delay.

Spermatozoa-like vessels, a distinctive vascular structure combining dotted and linear components, have been proposed as a highly specific dermoscopic feature of MF [24]. However, this morphology was not observed consistently or reproducibly in our study. Other authors have reported similar challenges in identifying this pattern [26]. The inconsistency in these findings may be attributed to the lack of standardized terminology for describing dermoscopic patterns in MF and the unavoidable inter-operator variability. This lack of uniformity complicates the identification of recurring patterns across studies, hindering reliable confirmation of their diagnostic value [21,22]. Standardizing dermoscopic descriptors could enhance pattern recognition and improve diagnostic accuracy. To clarify the specificity of these vascular morphologies in MF, comparative studies that analyze the dermoscopic features of MF against those of other dermatoses would be invaluable. Such research could determine whether these patterns are specific to MF or shared among other inflammatory or neoplastic conditions.

The variability in dermoscopic findings across studies may also be attributed to the heterogeneity of CLDs and their overlap with benign dermatoses. While our study did not identify significant correlations between dermoscopic features and the lesion type, other research has highlighted dermoscopy’s potential to reveal patterns, such as orange-yellowish patchy areas and white streaks, particularly in rarer clinical manifestations like poikilodermic MF [59,60]. The observation of brown pigmentation arranged in different configurations, such as dots, structureless areas, and reticular lines, was also associated with the poikilodermatous variant of MF.

Pigmentary changes appear to be the main appreciable features in MF dermoscopy in skin of color since the vascular changes are less detectable: the classic variant has been associated with white lines and a pseudo network resulting from brown-grey clouds and dots, while a weakened pigment network [38] characterizes the hypopigmented variant. However, no significant evidence about the reliability of dermoscopy in MF in skin of color is available [28].

These findings suggest that specific MF variants may exhibit distinct dermoscopic features, although further validation is needed.

Interestingly, a recent study focused on the trichoscopy of MF lesions observed a high prevalence of hair shaft abnormalities, such as numerous pili torti and 8-shaped hairs, compared to psoriasis and atopic dermatitis [31].

The limitations of this study include a small sample size, which may have hindered the detection of subtle correlations between dermatoscopic features and the disease stage. Additionally, the retrospective design and potential variability in image quality could have introduced bias. Inconsistencies in the terminology used to describe dermoscopic features may have further complicated the identification of recurring patterns, highlighting the need for standardized descriptors in future research. The potential interobserver variability in the evaluation of dermoscopic features is also important to consider: although all images were independently reviewed by two experienced dermatologists using standardized terminology, subjective interpretation cannot be entirely excluded. And, the dynamic evolution of dermoscopic patterns over time could not be assessed due to the cross-sectional nature of the dataset. Another study limitation is the lack of systematic correlation between dermoscopic features and histopathological findings. Due to the retrospective design, matched high-resolution histopathologic images were not consistently available for all cases and as a result, a direct side-by-side comparison of dermoscopic patterns with specific histological changes—such as folliculotropism, epidermotropism, or dermal fibrosis—could not be performed.

## 5. Conclusions

Our study highlights the limitations of using dermoscopy for diagnosing and staging MF and SS. While previous research has identified specific dermoscopic features with high sensitivity, our findings did not replicate these results, and further analysis revealed no significant correlation between dermoscopic features and the clinical stage or lesion type. This discrepancy suggests that the dermoscopic variability in MF may be more substantial than previously reported, potentially due to differences in imaging techniques, interobserver variability, and the retrospective nature of most studies.

Also, our dataset did not consistently observe hallmark features such as spermatozoa-like vessels, which were previously described to be highly specific for MF. This inconsistency may be attributed to heterogeneity in dermoscopic terminology and assessment criteria, which remain non-standardized across studies. The lack of a universal consensus on dermoscopic descriptors further complicates the reproducibility of findings and underscores the need for a systematic approach to dermoscopic feature classification in MF.

Despite the increasing interest in dermoscopy as a potential diagnostic tool for MF and SS, our results reinforce the necessity of clinical assessment and histopathological confirmation for an accurate diagnosis. The absence of reliable correlations between dermoscopic features and MF staging suggests that dermoscopy alone cannot replace standard diagnostic approaches, particularly in early-stage disease, where clinical and histopathological overlap with inflammatory dermatoses is common.

Future research should prioritize large-scale, multicenter prospective studies with standardized dermoscopic assessment criteria to improve diagnostic accuracy and interobserver agreement. Additionally, integrating artificial intelligence and machine learning models into dermoscopic analysis could enhance feature recognition and classification, providing a more objective and reproducible approach to MF diagnosis and monitoring. Until such advancements are established, dermoscopy should be considered a complementary tool rather than a definitive diagnostic method for MF and SS.

## Figures and Tables

**Table 1:** Demographic and clinical characteristics of the study population, including age distribution, sex, diagnosis, and disease stages.

Age		70 years	Std dev 14.56, CI. 59.29–69.71
Sex	Male	19	
	Female	11	
Diagnosis			
	Mycosis Fungoides	22	73.3
	Follicolotropic Mycosis Fungoides	4	13.3
	Sézary Syndrome	4	13.3
Stage	IA	7	23.3
	IB	8	26.6
	II	10	33.3
	III A	1	3.33
	IV A1	4	13.3

**Table 2:** Dermoscopy characteristics of the study population, detailing the presence of pigment, background color, vessel distribution, vessel types, scaling types, and keratin plugs. The table includes counts, percentages, and *p*-values to assess the statistical significance of these features.

Dermoscopy	Class	Value	Count	Percentage	*p* Value
	Pigment				<0.001
		Yes	0	0	
		No	30	100%	
	Background color				>0.05
		Red	17	56.7%	
		Orange	12	40%	
		Brown	1	3.3%	
	Vessel Distribution				>0.05
		Serpiginous	15	50%	
		Clustered	12	36.6%	
		Centered Dots	2	6.6%	
		Branched	1	3.3%	
	Vessel Type				>0.05
		Linear	12	40%	
		Serpentine	4	13.3%	
		Dots	11	36.7%	
		Clods	3	10%	
	Scaling Type				>0.05
		Diffuse Scaling	12	40%	
		Perifollicular Scaling	11	36.7%	
	Keratin Plugs				>0.05
		Yes	12	40%	
		No	18	60%	

**Table 3:** Summary of associations between dermoscopic features and lesion type or diagnosis. Fisher’s exact or Chi-square tests were applied as appropriate. Only vessel type showed a significant diagnostic correlation. Entries marked “N/A” refer to results with valid *p*-values but no additional informative value.

Test	Chi^2^ (df)	*p*-Value	Most Represented Pattern
Vessels Present vs. Diagnosis	0.38 (2)	0.829	—
Vessels Present vs. Lesion Type	4.14 (5)	0.530	—
Vessel Type vs. Diagnosis	20.79 (6)	0.002	Classical MF was most associated with linear vessels; FMF was most associated with serpentine vessels; and SS was most associated with dots
Vessel Type vs. Lesion Type	17.90 (15)	0.268	—
Vessel Distribution vs. Diagnosis	7.05 (6)	0.317	—
Vessel Distribution vs. Lesion Type	10.89 (15)	0.760	—
Keratin Plugs Present vs. Diagnosis	2.54 (2)	0.281	—
Keratin Plugs Present vs. Lesion Type	13.75 (5)	0.017	N/A
Background Color vs. Diagnosis	8.65 (4)	0.070	—
Background Color vs. Lesion Type	14.94 (10)	0.134	—
Scaling vs. Diagnosis	3.32 (2)	0.190	—
Scaling vs. Lesion Type	16.58 (5)	0.005	N/A
Localization Scaling vs. Diagnosis	5.57 (2)	0.062	—
Localization Scaling vs. Lesion Type	5.33 (4)	0.255	—

**Table 4:** Summary of studies analyzing the dermoscopic features of mycosis fungoides (MF), including study details, number of patients, identified dermoscopic patterns, and key finding.

Study	Journal and Article Title	Number of Patients	Dermoscopic Features	Findings and Relevance
Lallas et al. (2013) [23]	*Journal of the European Academy of Dermatology and Venereology*—‘Dermoscopy of early stage mycosis fungoides’	67	Fine short linear vessels, orange-erythematous background, and spermatozoa-like vessels	Fine short linear vessels (sensitivity: 93.7%; specificity: 97.1%) and orange-yellowish patchy areas (sensitivity: 90.6%; specificity: 99.7%) in early MF. A characteristic vascular structure resembling spermatozoa was also found to be highly specific for diagnosing mycosis fungoides.
Ozturk et al. (2019) [26]	*North Clinical Istanbul*—‘Dermoscopy of stage IIa mycosis fungoides’	17 (on 34)	Orange-yellow patches, short fine linear vessels, and geometric/perifollicular white scales are key markers for stage IIA MF diagnosis	Specific patterns can differentiate MF from PP, but spermatozoa-like structures, purpuric dots, collarette white scales, and Y-shaped arborizing vessels were observed but not statistically significant.
Nakamura et al. (2021) [28]	*Dermatology Practice & Concept*—‘Dermoscopy of Mycosis Fungoides and Its Variants in Patients with Skin of Color’	11	White streaks and pseudo-network in skin of color	Dermoscopic features of MF in patients with skin of color are predominantly characterized by striking pigmentary alterations. Vessel morphology is not a reliable diagnostic feature.
Errichetti et al. (2022) [25]	*Journal of the American Academy of Dermatology*—‘Dermoscopic spectrum of mycosis fungoides: a retrospective observational study by the International Dermoscopy Society’	118	Linear vessels and orange structureless areas, with a higher prevalence of patchy or furrow-aligned white scaling and linear-curved vessels	Dermoscopy can help identify classic MF, especially in the patch stage. Orange-yellow areas, spermatozoa-like vessels, and linear vessels aid in separating MF from dermatitis and psoriasis. Disease progression shifts from linear to branched vessels, with ulceration and bright white areas in tumors.
Soliman et al. (2023) [24]	*Dermatology Practical & Conceptual*—‘Dermoscopy in the Diagnosis of Mycosis Fungoides: Can it Help?’	88	Non-homogeneous pink to the erythematous background, patchy orange-red discolouration, whitish scales, and dotted, short linear, and spermatozoa-like vessels	Repetitive dermoscopic pattern in MF, including the cited dermoscopic features, with variations depending on the clinical variant.
Żychowska and Kołcz (2024) [27]	*Journal of Clinical Medicine*—‘Dermoscopy for the Differentiation of Subacute Cutaneous Lupus Erythematosus from Other Erythematous Desquamative Dermatoses’	26 (on 139)	Polymorphous vascular patterns include dotted, linear, and spermatozoa-like vessels, white/yellow scaling, and orange structureless areas. Variants may show perifollicular scaling, follicular plugs, and pigmentary changes	Dermoscopy aids in identifying classic MF, especially in the patch stage. Linear vessels and orange structureless areas are key features, while branched vessels and ulceration are seen in tumor-stage MF. Variant-specific features, such as follicular plugs in folliculotropic MF, can improve the diagnostic accuracy.
Jasińska et al. (2024) [31]	*Dermatology & Therapy*—‘Hair Shaft Abnormalities as a Dermoscopic Feature of Mycosis Fungoides: Pilot Results’	21 (on 55)	Hair shaft abnormalities (pili torti and 8-shaped hairs)	Hair shaft abnormalities are an important criterion that should be considered in the dermoscopic differentiation of patchy/plaque mycosis fungoides.
Mohamed Ali et al. (2025) [30]	*Archives of Dermatological Research*—‘Dermoscopy of Mycosis Fungoides: Could It Be a Confirmatory Aid to the Clinical Diagnosis?’	53	Fine short linear vessels, spermatozoa-like vessels, thick linear blood vessels, geometric white scales, white structureless patches, and orange-yellow patches	Linear vessels and brownish pigmentary changes suggest early-stage MF, while dotted vessels, purpuric dots, and ulcerations are linked to advanced MF. Geometric white scales and orange-yellow structureless areas lack specificity for stage.
Nasimi et al. (2021) [29]	*Australasian Journal of Dermatology*—‘Pigmented purpuric dermatoses versus purpuric mycosis fungoides: Clinicopathologic similarities and new insights into dermoscopic features’	28 (on 41)	Fine short linear vessels, spermatozoa-like structures, orange-yellow background, dotted vessels, erythematous globules, and reticular pigmentation	Fine short linear vessels and spermatozoa-like structures were significantly more common in purpuric MF, while PPD showed erythematous globules, reticular pigmentation, and a dull red background. Dermoscopy can aids in differentiating between these disorders

## Data Availability

Data available on request from the authors.
